# PCR amplification of *Bartonella koehlerae *from human blood and enrichment blood cultures

**DOI:** 10.1186/1756-3305-3-76

**Published:** 2010-08-24

**Authors:** Edward B Breitschwerdt, Ricardo G Maggi, B Robert Mozayeni, Barbara C Hegarty, Julie M Bradley, Patricia E Mascarelli

**Affiliations:** 1 Intracellular Pathogens Research Laboratory and the Center for Comparative Medicine and Translational Research, College of Veterinary Medicine, North Carolina State University, Raleigh, NC, USA; 2 Translational Medicine Group, Rockville, MD, USA

## Abstract

**Background:**

Cats appear to be the primary reservoir host for *Bartonella koehlerae*, an alpha Proteobacteria that is most likely transmitted among cat populations by fleas (*Ctenocephalides felis*). *Bartonella koehlerae *has caused endocarditis in a dog and in one human patient from Israel, but other clinically relevant reports involving this bacterium are lacking. Despite publication of numerous, worldwide epidemiological studies designed to determine the prevalence of *Bartonella *spp. bacteremia in cats, *B. koehlerae *has never been isolated using conventional blood agar plates. To date, successful isolation of *B. koehlerae *from cats and from the one human endocarditis patient has consistently required the use of chocolate agar plates.

**Results:**

In this study, *Bartonella koehlerae *bacteremia was documented in eight immunocompetent patients by PCR amplification and DNA sequencing, either prior to or after enrichment blood culture using *Bartonella *alpha Proteobacteria growth medium. Presenting symptoms most often included fatigue, insomnia, joint pain, headache, memory loss, and muscle pain. Four patients were also infected with *Bartonella vinsonii *subsp. *berkhoffii *genotype II. After molecular documentation of *B. koehlerae *infection in these patients, a serological test was developed and serum samples were tested retrospectively. *Bartonella koehlerae *antibodies were not detected (titers < 1:16) in 30 healthy human control sera, whereas five of eight patient samples had *B. koehlerae *antibody titers of 1:64 or greater.

**Conclusions:**

Although biased by a study population consisting of individuals with extensive arthropod and animal exposure, the results of this study suggest that *B. koehlerae *bacteremia is more common in immunocompetent people than has been previously suspected. Future studies should more thoroughly define modes of transmission and risk factors for acquiring infection with *B. koehlerae*. In addition, studies are needed to determine if *B. koehlerae *is a cause or cofactor in the development of arthritis, peripheral neuropathies or tachyarrhythmias in patients.

## Background

*Bartonella koehlerae *has a relative short microbiological history. In 1994, during a study designed to investigate the prevalence of *Bartonella henselae *bacteremia in domestic cats, *B. koehlerae *was isolated for the first time from the blood of two flea-infested healthy cats located on a farm in northern California [1,2]. Following experimental subcutaneous inoculation of one of these California *B. koehlerae *isolates, four cats became bacteremic for a mean of 74 days and each cat developed a species-specific antibody response to *B. koehlerae *outer membrane proteins [3]. Subsequently, *B. koehlerae *DNA was amplified from cat fleas (*Ctenocephalides felis*) collected from pets located throughout France [4]. Eighty-one of 309 fleas tested by polymerase chain reaction (PCR) and DNA sequencing contained a *Bartonella *spp.; *B. clarridgeiae *was found in 68%, *B. quintana *in 17%, *B. henselae *in 11%, and *B. koehlerae *in 4%. *Bartonella koehlerae *DNA was also amplified from an unidentified flea species removed from gerbils (*Meriones lybicus*) in Afghanistan [5]. *Bartonella koehlerae *was next isolated from a kitten in France suspected of having caused cat scratch disease in the owner [6]. Based upon these observations, cats are likely a primary reservoir host for *B. koehlerae*, as has been documented for *B. henselae *and *B. clarridgeiae*, with transmission among cats most likely occurring by infestations of *Ctenocephalides felis*; however, neither reservoir potential nor the mode of transmission have been definitively confirmed. To date, *B. koehlerae *has only been reported as a human pathogen in a single patient from Israel, who was diagnosed with culture-negative aortic valve endocarditis [7]. Those investigators subsequently isolated *B. koehlerae *from stray cats in Israel, which were the presumed source of infection for their patient. In 2010, *B. koehlerae *endocarditis was reported in a dog from Israel [8].

Historically, *B. henselae *and *B. clarridgeiae *have been frequently isolated from cat blood; however, successful isolation generally required prolonged incubation (weeks) in a high CO_2 _incubator. Using the same isolation approaches, *B. koehlerae *has been infrequently isolated, despite numerous, worldwide epidemiological studies designed to determine the prevalence of *Bartonella *spp. bacteremia in cats [9]. Therefore, it appears that *B. koehlerae *is more fastidious than *B. henselae *or *B. clarridgeiae*. To date, successful isolation of *B. koehlerae *from cats and the one human patient has consistently required the use of chocolate agar plates [1,2,7]. In recent years, our research group has focused on the enhanced diagnostic detection of *Bartonella *spp. in healthy and sick animals and in immunocompetent human patients [10-15]. Initial efforts to enhance the sensitivity of PCR detection of *Bartonella*-specific DNA sequences in patient samples, as a sole molecular diagnostic strategy, proved unsatisfactory. Therefore, we incorporated pre-enrichment culture of aseptically-obtained diagnostic specimens (blood, cerebrospinal, aqueous, and joint fluids and effusions) using a liquid insect cell culture-based medium (*Bartonella *alpha Proteobacteria Growth Medium, BAPGM) prior to PCR testing [10-16]. By combining enrichment culture followed by PCR amplification, diagnostic sensitivity was improved substantially over PCR alone, particularly when testing samples from sick dogs and immunocompetent human patients. The use of BAPGM has also facilitated the documentation of human infections with two novel *Bartonella *species [14,15].

In this study, we report the molecular detection of *B. koehlerae *DNA prior to or after enrichment culture in eight patients who were tested because of a spectrum of poorly defined illnesses. After molecular documentation of *B. koehlerae *infection in these patients, a serological test was developed and serum samples were tested retrospectively.

## Patients and Methods

Blood and serum samples from these individuals were submitted by an attending physician to the Intracellular Pathogens Research Laboratory (IPRL) for attempted isolation or molecular detection of a *Bartonella *species. In most instances, these patients were tested because of a history of extensive arthropod or animal contact. Patient samples were tested as a component of an IRB approved study entitled: Detection of *Bartonella *Species in the Blood of People with Extensive Animal Contact (North Carolina State University Institutional Review Board, IRB#s 4925-03 and 164-08-05). None of the patients reported in this study have been described in previous manuscripts from our laboratory [10,11,15]. A standardized questionnaire was completed by each individual and included age, gender, animal and arthropod exposure, outdoor activity, travel, clinical symptoms, duration of illness and co-morbid conditions. The duration of illness varied substantially among individuals, as did prior diagnostic evaluations and previous treatments.

The authors used a previously described approach incorporating pre-enrichment culture of blood in *Bartonella *alpha Proteobacteria growth medium (BAPGM) followed by PCR targeting the 16S-23 S intergenic spacer (ITS) region and the rpoB gene [10-13,16,17]. Bacterial isolation, PCR amplification, and cloning were performed using previously described methods [10,11,13,17]. Sequences were aligned and compared with GenBank sequences using AlignX software (Vector NTI Suite 6.0, InforMax, Inc.) [17]. The BAPGM diagnostic platform consists of PCR following direct extraction of DNA from both blood and serum samples, PCR following enrichment culture for at least 7 days, and PCR following subculture onto a blood agar plate, if colony growth is visualized. To assess for potential laboratory contamination, an un-inoculated BAPGM culture flask was processed simultaneously and in an identical manner with each batch of patient blood and serum samples tested. Specifically, while establishing cultures using a batch of samples in a biosafety hood, the top was removed from the BAPGM un-inoculated control flask until all patient samples had been processed. Following the standard operating procedures in the Intracellular Pathogens Research Laboratory, sample preparation including BAPGM cultures and agar plate sub-inoculation, DNA extraction, PCR preparation and PCR amplification and analysis were performed in separate laboratory rooms to avoid culture as well as DNA contamination. In addition, negative and positive Bartonella DNA test control samples, consisting of bacteria-free blood DNA and DNA spiked with *B. henselae *genomic DNA at 0.5 genome copies per microliter, respectively, were used for each batch of DNA tested. To minimize chance contamination with whole bacteria, the laboratory does not use a BAPGM positive control and has never used a *B. koehlerae *PCR positive control. All PCR and BAPGM culture negative controls remained negative throughout the duration of the study.

*Bartonella koehlerae, B. vinsonii *subsp. *berkhoffii, B. henselae and Rickettsia rickettsii *antibodies were determined following traditional immunofluorescence antibody assay (IFA) practices [10,11] with fluorescein conjugated goat anti-human IgG (Pierce Biotechnology, Rockford IL). *Bartonella vinsonii *subsp. *berkhoffii *genotype I (NCSU CO-1-93) and *R. rickettsii *(NCSU CO-1-08) originated from isolates made in our laboratory from natural canine infections. *B.koehlerae *(NCSU FO-1-09) and *B. henselae *H-1 (NCSU FO-93-23) were isolated from natural feline infections. Bartonella organisms were passed from agar grown cultures into AAE12 or DH82 cell cultures to obtain antigens as expressed when organisms are carried intracellularly in host blood. *R. rickettsii *cultures are maintained in cell lines due to the intracellular obligate growth requirements. Heavily infected cell cultures were spotted onto 30-well Teflon coated slides (Cel-Line/Thermo Scientific), air dried, acetone fixed and stored frozen. Serum samples were diluted in phosphate buffered saline (PBS) solution containing normal goat serum, Tween-20 and powdered nonfat dry milk to block non-specific antigen binding sites. All available patient sera were screened at dilutions of 1:16 to 1:64. All sera that were reactive at 1:64 were further tested with twofold dilutions out to 1:8192. To avoid confusion associated with possible non-specific binding found at low dilutions and to standardize with other laboratories such as the CDC, a cutoff titer of 1:64 was used to define a seroreactive titer. For preliminary testing purposes, *B. koehlerae *seroreactivity was assessed among six of the eight patients described in this study and using sera from 30 healthy people with minimal animal and arthropod exposure. A sufficient volume of serum was not available for 2 patients.

## Results

Between 2005 and 2009 the IPRL performed 430 human blood cultures using the BAPGM enrichment platform, of which 8 individuals were found to be infected with *B. koehlerae*. There were four males and four females, ranging in age from 24 to 64 years, all of whom resided in the eastern United States (Table 1). Four of the eight individuals in this study were veterinarians, all of whom had historical animal contact for at least 10 years. Of the non-veterinarians, patients 1 and 7 reported animal contact of at least 10 years, patient 7 reported 6 to 10 years of animal contact, whereas patient 5 reported no recent animal contact. All veterinarians reported bites or scratches from cats, dogs and less frequently other animal species. Overall, fatigue, insomnia, joint pain, headache, memory loss, and muscle pain were the most frequently reported symptoms (Table 2).

**Table 1:** Age, gender, state of residence and duration of illness in eight patients infected with *Bartonella koehlerae*.

Patient #	Age	Gender	Occupation	State of Residence	Duration of Illness
1	24	F	Manager, Retail Store	Pennsylvania	6 Months
2	59	F	Veterinarian	New Hampshire	6 Years
3	61	M	Veterinarian, Farmer	Florida	2 Years
4	41	F	Veterinarian	Virginia	18 Years
5	64	F	Homemaker	Maryland	9 Months
6	27	M	Artist	Maryland	2 Years
7	48	M	Landscape Architect	North Carolina	2 Months
8	33	M	Veterinarian	North Carolina	2 Months

**Table 2:** Symptoms reported by eight patients infected with *Bartonella koehlerae*.

Symptoms	Case 1	Case 2	Case 3	Case 4	Case 5	Case 6	Case 7	Case 8
Fatigue	X	X	X	X		X	X	X

Insomnia	X	X	X	X		X		X

joint pain	X	X	X	X	X		X	X

Headache	X	X	X	X		X		

Memory loss		X	X	X		X	X	

Sleepiness	X		X	X				

Muscle pain	X	X	X	X		X		X

Irritability		X	X	X		X		

Balance Problems		X	X	X		X		

Muscle weakness	X	X	X	X				

Blurred Vision		X	X			X		

Loss of sensation or numbness	X		X	X	X			

Shortness of breath			X	X				X

Confusion		X	X		X			

Eye pain			X			X		

Ventricular arrhythmia								X

Depression						X		X

Atrial Fibrillation					X			

Incontinence, urinary or bowel			X					

For each patient, 16S-23 S ITS region PCR results were confirmed by DNA sequencing (Table 3). As the ITS region varies among *Bartonella *species and strains, sequence length ranged from 425 to 670 base pairs. Subsequently, the *B. koehlerae *rpoB gene was successfully amplified and sequenced (660 bp) from six of eight patient blood samples. In the context of the BAPGM platform, *B. koehlerae *was more often amplified directly from patient blood or serum samples, whereas the *B. koehlerae *PCR was positive only after enrichment culture for 7 or 14 days in BAPGM for three patients. Only one patient was PCR positive following both direct extraction and after enrichment culture. No subculture isolates were obtained from any patient in this study. To date, the cat isolate used as a source of IFA antigen in this study is the only *B. koehlerae *subculture isolate obtained in our laboratory after BAPGM enrichment culture.

**Table 3:** Serological and PCR results for eight patients infected with *Bartonella koehlerae*.

Patient	Date	IFA Reciprocal Titers				PCR Results	
		***B. koehlerae***	***B. henselae***	***Bvb II***	***R. rickettsii***	**ITS PCR**	**rpoB PCR**

	10-2008	64	<16	<16	<16	Bvb*^,a^	Neg
1	02-2009**	<16	<16	<16	<16	*B. koehlerae*^a^	*B.koehlerae*^a^
1	04-2009**	<16	<16	<16	<16	Bspp^b^	Neg
							
2	03-2009	<16	<16	32	64	*B. koehlerae*^c^	*B.koehlerae*^a^
2	04-2009	NA	NA	NA	NA	Neg	Neg
2	07-2009**	<16	<16	<16	16	*B. koehlerae*^a^	Neg
2	10-2009**	<16	<16	<16	<16	Neg	Neg
							
3	03-2009	64	<16	64	<16	*B. koehlerae*^a^	*B.koehlerae*^a^
3	04-2009	NA	NA	NA	NA	*B. koehlerae*^c^	Neg
3	05-2009**	64	<16	32	<16	Neg	Bvb*^,a^
3	08-2009**	256	<16	16	<16	Neg	Neg
3	09-2009**	<16	<16	<16	<16	Neg	Neg
							
4	4-13-2009	<16	<16	<16	32	*B. koehlerae*^b^	Neg
4	4-30-2009**	<16	<16	<16	<16	Neg	Bvb*^,a^
4	6-02-2009**	<16	<16	32	<16	Neg	Neg
4	6-09-2009**	<16	<16	64	16	Neg	Neg
4	6-30-2009**	64	<16	64	<16	Neg	Neg
							
5	05-2009	NA	NA	NA	NA	*B. koehlerae*^a^	*B.koehlerae*^a^
							
6	05-2009	NA	NA	NA	NA	*B. koehlerae*^a^	*B.koehlerae*^a^
							
7	04-2009	64	<16	32	128	*B. koehlerae*^b^	Neg
7	06-2009	64	<16	64	64	Neg	Bvb*,^a^
7	07-2009	64	<16	<16	64	*B. henselae*^a^	Neg
7	11-2009**	256	<16	<16	16	*B. henselae*^a^	Neg
							
8	04-2009	64	<16	<16	16	*B. koehlerae*^b^	Neg
8	05-2009	64	<16	<16	<16	*B. koehlerae*^a^	*B.koehlerae*^a^
8	07-2009	64	<16	<16	32	Neg	Neg
8	08-2009	<16	<16	32	<16	Neg	Neg

All ITS and rpoB PCR amplicons were sequenced to confirm genus and species identity.

*Bvb = Bartonella vinsonii subsp. *berkhoffii *genotype II

Bspp = amplicon generated with 16S-23 S ITS primers, but unable to obtain a DNA sequence.

** Denotes post-antibiotic testing. NA = Sample not available.

^a ^= amplicon sequenced from blood or serum

^b ^= amplicon sequenced from BAPGM enrichment blood culture

^c ^= amplicons sequenced from both blood and BAPGM enrichment blood culture

By retrospective testing, *B. koehlerae *antibody titers ranged from <1:16 to 1:256 in the six patients, for whom serum was available. Five of these six patients had titers of 1:64 or greater (Table 3). *Bartonella koehlerae *antibodies were not detected (titers < 1:16) in the 30 healthy control sera. Three patients had a 1:64 IFA titer to *B. vinsonii *subsp. *berkhoffii *and DNA of this subspecies was sequenced from these 3 patients, whereas one patient was PCR positive for *B. vinsonii *subsp. *berkhoffii *and *B. henselae *at different testing time points, but was not seroreactive to *B. henselae*. Due to the history of repeated seroreactivity to *Rickettsia rickettsii *antigens in patient 7, all patient sera were tested for potential cross reactivity to *Rickettsia *antigens. IFA titers to *R. rickettsii *ranging from 1:64 to1:128 were found in two patients (#2 and #7) (Table 3).

A brief medical history for each patient follows:

**Patient 1 **was examined by a rheumatologist in October 2008 due to recent onset of fatigue, headache, insomnia and bilateral hip pain. This patient reported daily contact with dogs, no direct contact with other animal species and minimal to no exposure to arthropods. *Bartonella vinsonii *subsp. *berkhoffii *was amplified and sequenced after direct extraction from a blood sample; however, growth was not obtained by BAPGM enrichment culture. She was not seroreactive to *B. vinsonii *subsp. *berkhoffii *or *B. henselae *antigens, but was retrospectively found to be seroreactive to *B. koehlerae *antigens (Table 3). Treatment with minocycline was accompanied by more frequent headaches and increased hip pain. She then received azithromycin with some decrease in headache frequency, but no change in hip pain. Following antibiotic administration, she was no longer *B. koehlerae *seroreactive; however, in February 2009, due to persistent fatigue, headache, hip joint and muscle pain, and newly acquired numbness and tingling sensations in the feet and hands, the patient was retested in the IPRL. *B. koehlerae *was amplified following direct extraction from blood, but bacterial DNA was not amplified from the enrichment or subcultures. Shortly after adding rifampin to the azithromycin in March 2009 she experienced a Jarisch-Herxheimer-like reaction with acutely worsening symptoms; however, after continuation of both antibiotics there was gradual improvement in energy level, decreased hip pain and less frequent headaches during the subsequent 2 months. In November 2009, two months after completing antibiotics, the woman reported decreased amplitude and frequency of hip pain.

**Patient 2**, a veterinarian with extensive companion animal contact, reported an eight-year history of illness, which was primarily characterized by muscle pain. Fibromyalgia was diagnosed in March 2003. After *B. koehlerae *was amplified and sequenced from a BAPGM enrichment culture in March 2009, the woman was treated with a 10-week course of rifampin and levofloxacin. Forty eight hours after starting antibiotics, she experienced a Jarisch-Herxheimer-like reaction, after which there was gradual, but definite clinical improvement, characterized by decreased stiffness and muscle pain, particularly in the morning and after exercise. Eight weeks after completing the course of antibiotics the patient reported resolution of muscle pain and metatarsophalangeal joint pain, which was reportedly swollen for ten years.

**Patient 3**, also a veterinarian, was chronically ill for 2 years. He had extensive contact with a spectrum of pet and production animals, also frequently participated in outdoor activities, including farming, hiking and hunting and had daily to weekly exposure to fleas, ticks, biting flies, and mosquitoes. His initial symptoms were most consistent with a severe, protracted flu-like illness, accompanied by fatigue and headaches. Afterwards, he developed numerous other symptoms which would wax and wane in severity, including shooting pain originating at the base of the skull and radiating over the head (Table 2). The most serious symptom described by this patient was frequent to near constant coughing resulting in production of large quantities of green sputum that induced choking and difficulty breathing. Based upon allergy test results, the patient was hyposensitized for nearly one year, without symptomatic improvement. Chronic obstructive pulmonary disease (COPD) was diagnosed, but no microbiological testing was performed. Approximately 6 months after development of bronchial signs the man developed sinusitis, which in March 2008 required surgical drainage. The respiratory illness had become so debilitating that continuing to work was a daily challenge and eventually the man avoided meetings and social gatherings due to the disruptive nature of his cough. *B. koehlerae *DNA was amplified and sequenced following direct extraction from two blood samples obtained five weeks apart and for a third time from the initial BAPGM enrichment culture. After a 3-month course of doxycycline and rifampin, he experienced an increased energy level accompanied by substantial overall symptomatic improvement including resolution of the sinusitis, a nearly complete elimination of the cough and a notable decrease in right stifle joint crepitus, which he had previously attributed to old age. There was no relapse in symptoms during the eight month follow-up period.

**Patient 4 **developed retro-ocular headaches and pain in the elbow and shoulder in 1991, at the time of graduation from veterinary school. Within a year, pain also involved the hip joints and knees. Due to subsequent involvement of peripheral joints, accompanied by increasingly severe fatigue, the patient was cared for by a rheumatologist and various other medical specialists. Beginning in 2001, she developed a peripheral neuropathy. *B. koehlerae *DNA was amplified and sequenced from the initial BAPGM enrichment blood culture whereas *B. vinsonii *subsp. *berkhoffii *was amplified and sequenced directly from a post-treatment blood sample, obtained one month later. *B. koehlerae *DNA was concurrently amplified and sequenced from one of her pet cats. Following administration of doxycycline, *Bartonella *spp. DNA was not amplified from three subsequent blood culture attempts, but there was minimal change in symptoms. Beginning in August 2009, azithromycin and minocycline were prescribed for three months, during which time symptomatic improvement was reported. In March, 2010, the patient reported resolution in joint and muscle pain, peripheral neuropathy, neurocognitive abnormalities and proprioceptive deficits.

**Patient 5 **had a history of gastroparesis, paroxysmal atrial fibrillation and flutter that developed in December 2008 (status post ablation therapy), and a 2 year history of bilateral symmetric tingling in the first 3 toes of both feet with no loss of sensation, similar but milder neuropathy in her hands, and cognitive impairment, which she described as 'brain fog'. She had been treated medically for atrial fibrillation and osteoporosis. She reported minimal to no exposure to biting insects, no recent contact with animals, but had previously owned a dog. The patient had numerous small varicosities on the lower extremities, lower extremity bursitis; and tenderness in the right lateral quadriceps area. *B. koehlerae *was amplified directly from the blood sample but the BAPGM enrichment culture and subcultures were negative. Serum was not available for serological testing. In June 2009, azithromycin was administered for *B. koehlerae *infection. By July 2009 the woman reported improved cognition and short term memory, and increased energy. During the next 3 months, azithromycin was continued and the patient experienced increased symptoms of GI dysmotility, intermittent flu-like symptoms accompanied by intense chills without rigors and constant symmetric tingling of first 3 toes. Despite these symptoms, the "brain fog" and tingling in the hands were much improved, there was some improvement of neuropathy on left side, but the integrity of her short term memory fluctuated.

**Patient 6 **was diagnosed with idiopathic multi-focal retinitis by an ophthalmologist in August, 2008, which responded to high dose prednisone (60 mg/day). The patient subsequently developed cardiac arrhythmias, characterized by extrasystoles and pauses, skeletal muscle twitching and spasms, and fatigue in November 2008. A fluorescence angiogram again documented retinal inflammation and prednisone therapy was re-instituted. In June 2009, the patient reported partial overall improvement in symptoms while taking prednisone. A rheumatoid factor assay was positive (21, reference range 0-14). A vasculopathy, with accompanying retinopathy, was diagnosed.

There was no history of tick bites, but the patient had rescued three feral cats, which he maintained as pets and had experienced cat scratches and bites. *B. koehlerae *was amplified directly from the blood sample, but the BAPGM enrichment culture and subculture were negative. The patient was treated with azithromycin and minocycline. When examined in December 2009, he reported overall symptomatic improvement, despite having not completed the entire course of antibiotics. Treatment with azithromycin and rifamipin for the next six months resulted in alleviation of symptoms, including no recurrence of visual floaters and light flashes.

**Patient 7**, who had daily dog contact and participated frequently in outdoor activities, reported recent onset of severe fatigue, difficulty remembering and joint pain and a history of tick exposure. During the past few years the patient developed a dietary allergy to beef and pork and fatigue, aches, "fogginess" that he had attributed to the aging process. Antibodies to *Rickettsia rickettsii *were previously detected by a commercial laboratory and antibodies were also detectable in the IPRL in three serum samples obtained at monthly intervals. This individual was consistently seroreactive to *B. koehlerae *antigens, intermittently seroreactive to *B. vinsonii *subsp. *berkhoffii*, but was never found to be seroreactive to *B. henselae *(Table 3). *Bartonella koehlerae, B. henselae *or *B. vinsonii *subsp. *berkhoffii *were amplified directly from four blood samples obtained over a seven-month time period. After a three-month course of doxycycline and rifampin, *B. henselae *DNA was again sequenced from an extracted blood sample in November 2009 and the *B. koehlerae *titer had increased from 1:64 to 1:256. During the months following antibiotic administration the patient reported gradual improvement in symptoms and by February 2010 he described a significant improvement in the "fogginess" and improved ability to remember and decreased joint pain.

**Patient 8 **The blood culture from Patient 8 was obtained due to the acute onset of ventricular tachyarrhythmia's that developed three weeks after coccygodynia surgery and during a professionally stressful period in this individual's life. Concurrently, this patient reported difficulty breathing, palpitations and anxiety. Other medical problems reported during the previous four years, included bilateral knee pain with patellar chondromalacia, documented by MRI, bilateral carpal tunnel syndrome and tinnitus involving the left ear. Although a veterinarian, with extensive prior animal contact, this individual had minimal occupational exposure during the 4-year period prior to PCR detection of *B. koehlerae *in the initial BAPGM enrichment blood culture. A blood sample obtained one month later also contained *B. koehlerae *DNA. As two subsequent blood cultures were *B. koehlerae *negative and because the patient was no longer *B. koehlerae *seroreactive, this individual elected to not be treated with antibiotics.

## Discussion

Although biased by a study population consisting of individuals with extensive arthropod and animal exposure, the results of this study suggest that *B. koehlerae *bacteremia occurs more frequently (8 of 430 patients tested, 1.8%) in immunocompetent patients than previously suspected. Also, as the duration of illness in these patients ranged from two months to eighteen years, *B. koehlerae *may induce a chronic intravascular infection, as recently proposed for *B. vinsonii *subsp. *berkhoffii *and *B. henselae *[18]. In addition, repeated amplification of *B. koehlerae *DNA from sequentially obtained blood samples from patients 2, 3, and 8 further supports the possibility of a persistent or relapsing pattern of bacteremia. Similar to two previous studies [10,11], the mean age of this patient cohort was 45 years. The most frequent symptoms reported by these patients included fatigue, insomnia, joint pain, headache, memory loss, and muscle pain. Although non-specific, these symptoms are very similar to those previously reported in bacteremic patients that were infected with *B. henselae, B. vinsonii *subsp. *berkhoffii*, Candidatus *B. melophagi *or individuals co-infected with more than one *Bartonella *spp. [10,11,15]. Because recent studies describe very similar patterns of illness in immunocompetent patients [10,11,15], prospective controlled studies are needed to critically assess whether there is an association between chronic intravascular infection with *Bartonella *spp. and specific disease entities. Importantly, sequential studies involving veterinarians and veterinary technicians, in conjunction with appropriate control groups, might allow for more rapid determination of the pathogenic potential of a growing number of zoonotic *Bartonella *spp. in immunocompetent individuals.

Our findings do not confirm that *B. koehlerae *was a cause or co-factor in the illnesses described in these patients or that the response to therapy was related to elimination of this bacteria. However, despite protracted medical histories, all seven patients treated with antibiotics reported substantial symptomatic improvement and in most instances *B. koehlerae *antibodies and DNA were no longer detected in post-treatment samples. Failure to consistently detect *B. koehlerae *bacteremia by repeat testing in patients 2 and 7 prior to antibiotic administration or in patient 8 who was never treated with antibiotics could relate to a relapsing pattern of bacteremia, as demonstrated following experimental inoculation of *B. henselae *and *B. clarridgeiae *into cats [19]. Alternative explanations include limited sensitivity of the BAPGM platform for the detection of *B. koehlerae*, or immunological clearance of these bacteria from the vasculature. Although co-infection with more than one *Bartonella *sp. has been reported with increasing frequency in human patients [10,11,18,20], the detection of *B. vinsonii *subsp. *berkhoffii *DNA in four of eight patients infected with *B. koehlerae *was an unexpected finding. Cats are considered the primary reservoir hosts for *B. koehlerae*, whereas dogs and wild canines are the primary reservoir hosts for *B. vinsonii *subsp. *berkhoffii*. Infection with *B. vinsonii *subsp. *berkhoffii *spanning an eighteen month time frame occurred in a cat with recurrent osteomyelitis [21]. Although the mode of *B. vinsonii *subsp. *berkhoffii *transmission could not be determined, patients 3 and 4 were companion animal veterinarians, who had frequent contact with both cats and dogs; whereas, patients 1 and 7 reported primarily dog contact.

When attempting to establish if a patient has been previously exposed to or is actively infected with *B. koehlerae*, serology, direct PCR from blood and serum and enrichment culture should be examined. Although IFA results reported in this study should be considered preliminary, five of six patients for whom serology was performed were *B. koehlerae *IFA seroreactive at titers of 1:64 to 1:256, whereas *B. koehlerae *antibodies were not detected in thirty healthy individuals. There was no serological crossreactivity to *B. henselae *antigens in these *B. koehlerae *infected patients. As reported for other *B. henselae *infected individuals [10,11], patient 7 was never *B. henselae *seroreactive, despite sequencing *B. henselae *DNA at two temporally separated sample testing time points. In contrast to high antibody titers and serological crossreactivity reported in patients with *Bartonella *sp. endocarditis, these bacteremic patients had low antibody titers and minimal crossreactivity. Previous studies have suggested the possibility of serological cross reactivity between *Bartonella *and *Rickettsia *spp. antigens [22]. Patients 2 and 7 had *R. rickettsii *antibody titers ranging from 1:64 to 1:128, whereas patients 4 and 8 had very low titers (1:16 and 1:32 at different time points). There was no consistent pattern of cross reactivity among *Bartonella *and *Rickettsia *antigens in this study, therefore prior exposure to a *Rickettsia *sp. seems more likely than crossreactivity among *Rickettsia *and *Bartonella *sp. antigens. When specific pathogen free dogs were infected with *B. vinsonii *subsp. *berkhoffii *genotype I, there was no cross reactivity to *R. rickettsii *antigens by IFA testing [23].

The medical history of patient 3, an older veterinarian, was unique in the context of severe, chronic bronchitis and sinusitis. The pulmonary disease was of such a severity that the man could only marginally function in routine work related activities. Atypical manifestations and more severe clinical presentations have been proposed to occur in older individuals infected with *Bartonella *spp. [24]. Although considered infrequent, pulmonary manifestations have been associated with *B. henselae *infections in patients with CSD [25]. In addition, *B. henselae *DNA was amplified and sequenced from nodular granulomatous pulmonary nodules that developed in a renal transplant recipient, who had extensive cat contact [26].

Patient 4 developed a peripheral neuropathy after a ten-year history of joint pain, whereas patients 1 and 6 reported tingling sensations in the hands or feet, which could be indicative of a sensory neuropathy. A distal axonal sensomotor polyneuropathy, vasculitis and Raynaud's phenomenon were reported in a 40 year-old man with a five year history of recurrent, painful toe ulcers [27]. Diagnosis of bartonellosis in that patient was supported by a *B. henselae *antibody titer of 1:1,024 and remission of the vasculitis and the polyneuropathy following treatment with erythromycin for three months followed by doxycycline for three weeks. Biopsy of the patient's sural nerve demonstrated an axonal neuropathy with a neurogenic muscular atrophy. The authors concluded that *B. henselae *infection should be considered in patients with vasculitis and polyneuropathic syndromes. It is also possible that *B. koehlerae *should also be among the differential diagnoses for patients with peripheral neuropathy.

Interestingly, three patients in this study reported atrial or ventricular tachyarrhythmias. Although an increasingly well recognized cause of endocarditis, the potential role of *Bartonella *spp. as a cause of myocarditis or arrhythmias has been incompletely studied [28]. *Bartonella henselae *has been implicated on the basis of molecular pathology as a cause of sudden death in Swedish orienteers [29]. In a more recent study from Switzerland, arrhythmogenic right ventricular cardiomyopathy (ARVC), an important cause of sudden death in young adults, was serologically associated with *B. henselae *antibodies [30]. These authors concluded that further studies in larger patient cohorts seem justified to investigate a possible causal link between chronic *B. henselae *and ARVC, in particular the sporadic (nonfamilial) form of the disease. Chronic active myocarditis requiring heart transplantation was also reported in a previously healthy young man after developing *B. henselae*-induced cat scratch disease [31]. In the context of intravascular pathogens, *Bartonella *species may prove to be unique, as these bacteria can induce intraerythrocytic infection, vascular endothelial infection and experimentally can achieve intracellular localization within monocytes, macrophages, dendritic cells and CD 34+ progenitor cells [32,33]. Assuming persistent infection coupled with a highly adapted ability to evade immune recognition by the host, these highly fastidious intravascular and intracellular bacteria could contribute to a spectrum of disease pathology over a protracted period of time [32].

On a daily basis, veterinarians are repeatedly exposed to a spectrum of animal species and to their associated arthropods. Unfortunately, due to the unknown duration of *B. koehlerae *infection in an immunocompetent person, the potential for repeated exposures over time and technical limitations associated with the molecular documentation of co-infection, the source and the timing of Bartonella infection in these individuals could not be accurately determined. Although veterinary professionals appear to be at a high risk of occupational exposure for *Bartonella *spp., numerous people in society have frequent or episodic exposure to animals and arthropod vectors. Increased attention to modes of transmission and risk factors for *Bartonella *spp. infections is warranted [34].

## Competing interests

In conjunction with Dr. Sushama Sontakke and North Carolina State University, Dr. Breitschwerdt holds U.S. Patent No. 7,115,385; Media and Methods for cultivation of microorganisms, which was issued October 3, 2006. He is the chief scientific officer for Galaxy Diagnostics, a newly formed company that provides diagnostic testing for the detection of *Bartonella *species infection in animals and in human patient samples. Dr. Ricardo Maggi performed all molecular microbiological testing reported in this study and is the Scientific Technical Advisor and Laboratory Director for Galaxy Dx.

## Authors' contributions

EBB was involved in all aspects of this study, including generation of the initial draft of the manuscript; RGM and PEM performed all blood cultures, PCR, DNA sequencing and molecular data analyses, BRM was responsible for patient evaluations, medical record review and patient follow-up, BCH and JMB were responsible for serological testing. All authors contributed to the content and approved the final manuscript.
